# Effect of Chinese Corporate Average Fuel Consumption and New Energy Vehicle Dual-Credit Regulation on Passenger Cars Average Fuel Consumption Analysis

**DOI:** 10.3390/ijerph18147218

**Published:** 2021-07-06

**Authors:** Haoyi Zhang, Fuquan Zhao, Han Hao, Zongwei Liu

**Affiliations:** 1 State Key Laboratory of Automotive Safety and Energy, Tsinghua University, Beijing 100084, China; haoyi-zh18@mails.tsinghua.edu.cn (H.Z.); zhaofuquan@tsinghua.edu.cn (F.Z.); Hao@tsinghua.edu.cn (H.H.); 2 Tsinghua Automotive Strategy Research Institute, Tsinghua University, Beijing 100084, China; 3 MIT Sloan School of Management, 77 Massachusetts Ave., Cambridge, MA 02139, USA

**Keywords:** new energy vehicle, dual-credit regulation, fuel consumption

## Abstract

The large sales volume and a great number of passenger car ownership in China have brought a series of environmental and energy problems. In response to these problems, Corporate Average Fuel Consumption and New Energy Vehicle Dual-credit Regulation has been put forward in China. However, it is found that although the purpose of the Dual-credit Regulation is controlling the fuel consumption and promoting the development of the energy vehicle market, the fuel consumption restriction for fossil-fueled passenger cars is relaxed compared to CAFC (Corporate Average Fuel Consumption) regulation alone. Moreover, this effect of relaxation is more obvious when the market share of new energy vehicles increases. To quantitatively estimate the relaxation effect of the fuel consumption restriction, a method of quantifying the relaxation effect is designed, and three different scenarios of new energy vehicle market development have been presumed in this paper. It is found that there are three main factors related to new energy vehicles that cause the relaxation of fuel consumption restriction, and the effect might become obvious and severe after 2025 if the market share of new energy vehicles develops very rapidly. These results may affect the development of the automotive industry and needed to be concerned.

## 1. Introduction

It was known that the automotive industry occupies a crucial position in China’s national economy. The growth of the automobile industry provides strong support for the sustainable development of China’s economy [1]. The auto market has been growing rapidly in recent years [2]. China exceeded the US in vehicle sales in 2009, becoming the largest vehicle market in the world [2]. Since then, China has remained the world’s largest vehicle market for 10 years [3]. According to the data of the China Association of Automobile Manufacturers, 25.769 million automobiles were sold in China in 2019. On the other hand, the rapid development of the automobile industry has also led to increasing oil consumption in China [4]. In 2019, 70.8% of China’s oil consumption depended on imports [5]. The development of energy-saving technologies is related to national energy security [6]. Since automobiles are the main source of fuel consumption [7], it is more necessary to control the fuel consumption of passenger cars, especially in recent years. The passenger car market continues to develop, and the sales of passenger cars reached 22.351 million in China in 2018 [8]. The high level of car ownership also results in high carbon emission, but the Chinese government had signed “The Paris Agreement,” which promised that the carbon emission in China would reach the peak before 2030. Therefore, the increasingly tightened fuel consumption regulations for passenger cars are an inevitable strategic choice for China, although it is hard for some companies [9]. At the same time, new energy vehicles are also a way to reduce fuel consumption. Therefore, the regulation promoting new energy vehicles will have far-reaching effects on reducing oil consumption and greenhouse gas emissions in the future [10]_._ Therefore, in September 2017, the Ministry of Industry and Information Technology and other departments officially relaxed the “Parallel Management of Passenger car Corporate Average Fuel Consumption and New Energy Vehicle Credit,” which is parallel management of credit for CAFC (Corporate Average Fuel Consumption) and NEV (New Energy Vehicle) [11] in order to reduce the fuel consumption of the ICEV (Internal combustion engine vehicle) and promote the development of the NEV market.

The current stage of the Dual-credit Regulation (2021–2023) was published in July 2019. However, the calculation method of CAFC credit and the mechanism of unidirectional compensation that NEV credit can compensate for the lack of CAFC credit leads to a negative impact on the fuel consumption controlling effect. This phenomenon may have a certain negative impact on the development of fuel-saving technologies. Therefore, a quantitative analysis of this impact is necessary. The results will be meaningful when designing the next stage of the CAFC regulation.

## 2. Brief of Dual-Credit Regulation

The United States took the lead in setting the CAFE (Corporate Average Fuel Economy) vehicle fuel consumption standard in 1975 [12]. Then, Japan (1979) and Europe (1980) established their own fuel consumption regulation [13]. In 2004, China promulgated the first mandatory standard for controlling automobile fuel consumption; it has continued to develop the standard and established the fourth-stage CAFC (corporate average fuel consumption) regulation in 2016. All these countries set stricter fuel consumption standards year by year to ensure that the national goals of passenger car fuel conservation could be met [14]. The technological strategy is important for companies to comply with the regulation. An OEM (Original Equipment Manufacturer) needs to optimally select several sets of fuel-efficient technologies to its assortment [15].

The Chinese Dual-credit Regulation consists of CAFC regulation and NEV regulation; each is issued for a different purpose. In order to reduce the average fuel consumption of passenger cars, China issued the first mandatory standard for controlling automobile fuel consumption in 2004, and three stages were implemented by 2015. Since 2016, China has implemented the fourth phase of the CAFC regulation for passenger cars. Compared with the third phase, the fuel consumption target value has been comprehensively tightened to further reduce the fuel consumption [16]. The main purpose of CAFC regulation is to enhance energy-saving technology and reduce fuel consumption. Since the implementation of the first stage of the fuel consumption regulation in China, the average fuel consumption of passenger cars has been 7.97 L/100 km based on the fuel consumption data released by the government [17], and the average fuel consumption of passenger cars in China has improved by 14.7% in the past eight years, according to a report published by the Innovation Center for Energy and Transportation in 2017 [18].

**Figure 1:**
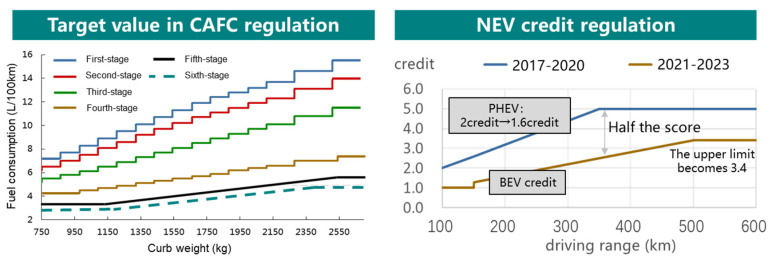
Target value in CAFC (Corporate Average Fuel Consumption) regulation and the NEV New Energy Vehicle) credit regulation. PHEV: plug-in hybrid vehicle; BEV: battery electric vehicle.

NEV regulation is the first mandatory regulation with the requirement of NEV sales in the world. A ratio requirement for NEV credits is set to make companies produce enough NEVs. Government can gradually raise the requirement of this ratio to promote the NEV market. The target value in CAFC regulation and the NEV credit regulation is shown in Figure 1. The target value curve of the sixth stage CAFC is presumed based on the previous stage. First, the target value curve of the fifth stage is shifted downward by 20% and then rotated to be more favorable for small cars.

According to the Dual-credit Regulation, the number of CAFC credits is given based on corporate average fuel consumption and target value. The corporate average fuel consumption is calculated by curb weight, fuel consumption and sales volume of the model. The target value of a corporation is the average target value of all vehicles, which depends on which mass segment the vehicle belongs to. The NEV credit of a corporation is the difference between the total NEV credit and the target value of NEV for this corporate. Among them, the NEV credit for each vehicle is calculated by basic credit and a multiple. The basic credit for each BEV will be significantly reduced by about half. The basic credit for each PHEV is reduced from 2 to 1.6, as shown in Figure 1. The multiple of a NEV depends on its power consumption.

Besides, there are ordinates including a unidirectional compensation mechanism that the lack of CAFC credit can be compensated by positive NEV credits. Ordinates like this cause an effect on fuel consumption restriction.

There are strong incentives for the application of fuel economy technologies and the development of the NEV market in the current stage of Dual-credit Regulation. Companies should pay attention to the development of fuel economy technologies [19] and lay out the development of NEVs. However, corporations benefit from the average fuel consumption calculation process in the regulation because of the NEV, including the battery electric vehicle (BEV), plug-in hybrid vehicle (PHEV), and fuel cell vehicle (FCV). Not only the fuel consumption of NEVs is calculated to zero, but also an advantage multiple is given when calculating the corporate average fuel consumption [20]. These preferences make the production of NEVs conducive to regulation compliance. Moreover, multiple compliance strategies are provided in the fourth-stage regulation. Companies are allowed to compensate for the lack of CAFC credits with NEV credits, which means some companies can comply by producing or purchasing NEV credits, and the difficulty of compliance is further reduced.

## 3. Literature Review

There is a lot of research about CAFC and NEV regulation. There is a large number of research studies about the influence of Dual-credit Regulation on NEV production [21,22]. Consensus has been reached that the Dual-credit Regulation conducive to promoting the development of NEV. Ou’s research shows a positive effect on PHEV [23].

Li Y. analyzed the influence of Dual-credit Regulation on the ICEV and NEV production [24], while Lou et al. (2020) analyzed the production and fuel consumption of ICEVs, these researches study from the perspective of economic interest of enterprises base on parameters like prices of vehicles and NEV credits.

From Yu’s research studies about the substitution effect of Dual-credit Regulation and the NEV credit trading platform on subsidies for NEV, it is known that the changes of Dual-credit Regulation rules may have different effects on the optimal production and pricing strategies in different CAFC cases [25]. Moreover, Wang studies the automakers’ strategies for meeting the Dual-credit Regulation [26]. While Zhao analyzed the positive effect on technology development for electric vehicles under the Dual-credit Regulation [27].

These research studies about the Dual-credit Regulation from different perspectives, including the technology roadmap, economic benefits of enterprises, market distribution and the substitution effect of subsidies. However, there is a lack of research about the control effect on the passenger car fleet fuel consumption regulation caused by the regulation itself, especially the latest stage Dual-credit Regulation. Since this unknown effect will hinder the government’s control over the average fuel consumption of the fleet, it must be studied so that the government has effective means to control the average fuel consumption of the fleet more accurately.

## 4. Research Methods

This paper mainly studies the impact of NEVs on the fuel consumption control effect under the Dual-credit Regulation. In the Dual-credit Regulation, the restriction of the fuel consumption has been relaxed mainly because of these three factors:

(i) Special calculation method for NEVs in the CAFC regulation, and there are two factors that related to the relaxation;

(ii) NEVs are accounted for higher multiples when calculating the average fuel consumption in the CAFC regulation so that the CAFC value of an enterprise would be lower than the actual value and make complying with the Dual-credit Regulation easier;

(iii) The fuel consumption is regarded as zero in the CAFC regulation. Therefore, the calculated average fuel consumption value is lower than the actual value. Since the fourth stage of the CAFC regulation is drafting, a lot of studies have been carried out to discuss the conversion method of the NEV electric consumption; three methods are mentioned: equal caloric, fuel life cycle (FLC), and carbon emission. Although “regard as zero” is still adopted, details about other conversion methods have not been released, but experts from China Automotive Technology and Research Center (CATARC) have a consensus on the fact that “regard as zero” is too low. According to their preliminary investigation, BEV with a power consumption of 14.3 kWh/100 km corresponds to 1.66 L/100 km under equal caloric method, 3.20 L/100 km under the FLC method and 3.77 L/100 km under carbon emission method. In this paper, the fuel consumption of NEVs is presumed to be converted on the basis of equal caloric value from 2025 onwards, and the coefficient is assumed to be 0.116 L/kWh.

**Figure 2:**
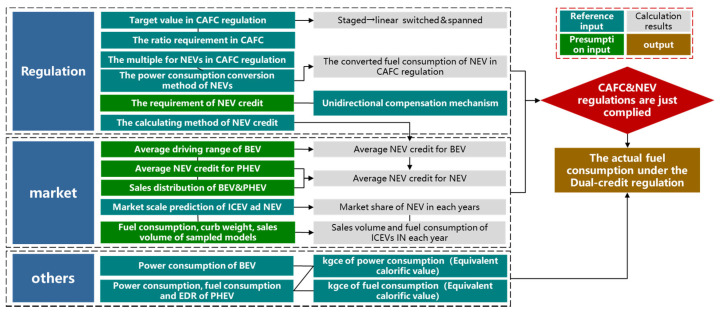
The framework of the calculation method. ICEV: Internal combustion engine vehicle; EDR: Electric Distance Ratio; CAFC: Corporate Average Fuel Consumption.

In order to visually quantify the effect of the relaxation, the basic research method is to calculate the NEV credit under different NEV market distributions and then find out the highest average fuel consumption value of the ICEV fleet when the Dual-credit Regulation just complies. Then the difference between this value and the target value in CAFC regulation can quantify the degree of the relaxation. The logic between all relevant parameters is shown in Figure 2. To begin with, the following basic assumptions have been made for the passenger car market:

(i) The NEV credit transaction is complete, the needs of each company for credit can be satisfied;

(ii) Each company can just comply with the Dual-credit Regulation after the NEV credit transaction, and there is no remaining credit;

(iii) The average curb weight of the ICEV fleet does not change over time, and the sales distribution of different curb weight segments between NEV and ICEV is the same.

These assumptions make it possible to quantify the relaxation effect of the fuel consumption-controlling Dual-credit Regulation on the fossil-fueled vehicle.

In this paper, the average NEV credit for each NEV on average is presumed base on the industry average point of view, and the method of this presumption is described in Section 5. The target value of the CAFC for every corporation is based on statistics of 14 companies, which are the top seven Chinese companies and the top seven foreign companies in sales volume. The data is listed in the appendix, but the name of these enterprises and models are removed to protect the confidentiality of these enterprises.

The corporate average fuel consumption is calculated as Equation (1), considering the sampling rate in this research. The target value is calculated as Equation (2). These two equations are formulated according to the CAFC regulation. The actual fuel consumption is calculated as Equation (3). The actual average fuel consumption after 2018 is calculated based on the fuel consumption and progress rate of the fuel consumption in the previous year, as shown in Equation (4). The progress rate in Equation (4) will make this company comply with the Dual-credit Regulation in the next year.

(1) $$C_{CAFC}=\frac{\sum_{i=1}^{n}\frac{FCf_{i}\times vf_{i}}{r}+FC_{BEV}\times v_{BEV}+FC_{PHEV}\times v_{PHEV}}{\frac{vf_{i}}{r}+W_{y}\times \left(v_{PHEV}+v_{BEV}\right)}$$

(2) $$T_{CAFC}=\frac{\sum_{i=1}^{n}T_{i}\times vf_{i}}{\sum_{i=1}^{n}vf_{i}}$$

(3) $$FC=\frac{\sum_{i=1}^{n}FCf_{i}\times vf_{i}}{\sum_{i=1}^{n}vf_{i}\times r}$$

(4) $$p_{y}=\frac{T_{NEV}-F_{NEV}+\frac{\sum_{i=1}^{n}T_{i}\times Vf_{i}}{\sum_{i=1}^{n}Vf_{i}}\sum_{i=1}^{n}W_{i}\times vf_{i}\times r}{\sum_{i=1}^{n}FC_{i}\times vf_{i}}$$

In the equations above: *C_CAFC_* is the calculated corporate average fuel consumption; *FCf_i_* is the fuel consumption of the *i*-th sampling fossil-fueled vehicle model; *FC_BEV_* is the average conversion fuel consumption of battery electric vehicles; *FC_PHEV_* is the average conversion fuel consumption of plug-in hybrid vehicles; *vf_i_* is the sales volume of the *i*-th sampling fossil-fueled vehicle model; *v_BEV_* is the sales volume of the battery electric vehicle; *v_PHEV_* is the sales volume of the plug-in hybrid vehicle; *W_y_* is the multiple of the NEV in CAFC regulation; *r* is the sampling rate of the fossil-fueled vehicle; *T_i_* is the target value of the *i*-th vehicle model; *p_y_* is the rate of fuel consumption progress in the year *y*; *T_NEV_* is the Minimum ratio requirement of NEV credit (the ratio of NEV credit to the fossil-fueled vehicle sales volume); *F_NEV_* is the NEV credit for the year.

Some of the provisions are not considered in these formulas, such as the preferential provisions for fuel-saving vehicles, which are added in the fifth stage of the regulation. For example, models with lower fuel consumption than their target value will be regarded as 0.5, 0.3, and 0.2 when calculating the target value of NEV credit and make it easier to comply with the NEV credit regulation for companies.

According to the *T_CAFC_* and *C_CAFC_*, the CAFC credit for the company can be determined. Then, the relaxation degree can be quantified. Moreover, the effect of the three factors mentioned above can also be quantified.

## 5. Market and Regulation Hypothesis

Parameters about the Dual-credit Regulation, Chinese passenger car market, and NEV technology before 2030 are presumed in this paper for analysis.

### 5.1. Passenger Car Market Hypothesis

The passenger car market hypothesis is mainly based on the development of passenger car sales in recent years and the “Technology roadmap for energy-saving and NEVs” (hereinafter referred to as “The Roadmap”) [28]. The following parameters are presumed: the proportion of NEVs sales volume in passenger car sales, the sales distribution of the BEVs and PHEVs, the average power consumption and fuel consumption per 100 km of BEVs and PHEVs. Three scenarios are set, “L” indicates the development of the NEV market can make it just fulfill the minimum ratio requirement of new energy points; “M” and “H” indicates the upper and lower proportion of NEVs in car sales, predicted in “The Roadmap” [28]. It is predicted that the proportion of NEVs will rise quickly and will reach a ratio between scenario “M” and “H” because the growth of the car market in China is slowing down apparently, and NEVs started to show strong potential for growth. In addition, due to the relatively stable demand for commercial vehicles, the demand and sales volume are presumed to be unchanged in recent years. The NEV sales proportion in some years is predicted and shown in Table 1. The proportion between these predicted years is calculated to keep the growth rate between them unchanged.

In the Dual-credit Regulation, the NEV can be divided into two types: BEV and PHEV. The NEV credit is given in different principles. Moreover, the calculation methods of the actual power consumption and the fuel consumption are different between these two kinds of NEVs. Therefore, it is necessary to presume the sales ratio of the NEVs, and then based on this ratio, the average credit of a NEV in the market can be inferred. The sales ratio of plug-in hybrid vehicles showed a downward trend from 2015 to 2017. However, plug-in hybrid vehicles have certain advantages in terms of the technical level. For example, parallel sources of relative ease with which the useful range of the vehicle can be extended by providing an internal combustion engine that can be used for battery charging as well as for power boosting under acceleration or load conditions [29]. Moreover, in terms of subsidies, including manufacturing cost and convenience, PHEV also has certain advantages. These advantages have led to an increase in sales. Moreover, the ratio of plug-in hybrid models may rebound slightly after 2020, and finally, the sales ratio of battery electric vehicles and plug-in hybrid vehicles will remain at about 2:1. The distribution of the NEV sales, the average credit for one battery-electric vehicle, the credits of the plug-in hybrid vehicle, and the average NEV credits for NEV are shown in Table 2. Further, the data of other years is presumed by exponential interpolation.

The fuel consumption data of both fossil-fueled and NEV is needed in the process of calculating the CAFC credit. To deal with this problem, the average conversion fuel consumption data of NEV from 2018 to 2030 is presumed, mainly based on the data of 2016. It is known that there are two operation modes when PHEV is driving, which are the charge depleting mode (CD) and the charge sustaining mode (CS). To make better presumptions, a parameter called EDR (Electric Distance Ratio) is used in this paper, as shown in Table 3. The typical Class A PHEV models will have a fuel consumption of less than 5 L/100 km, 4.5 L/100 km, and 4.3 L/100 km in the CS mode at 2020, 2025, and 2030, as proposed in “The Roadmap” [28]. Respectively, the prediction of BEV power consumption is also based on “The Roadmap,” that the power consumption should lower than 13 kWh/100 km, 11 kWh/100 km, and 10 kWh/100 km in CD mode at 2020, 2025, and 2030. Since the average curb weight of the PHEV in the market is higher than the A-class, the energy consumption in 2020 and 2025 is converted by 1.2 times of the 2020 value in “The Roadmap” and 1.3 times in 2030. The power consumption and the fuel consumption of BEV and PHEV should decrease by 10% every five years [28]. It is presumed that the conversion method will change into an equal caloric value method after 2025. Finally, the comprehensive energy consumption will be obtained base on Equation (5), and the results are adjusted based on the actual data collected from the Chinese car market, 2016–2020. The data for other years are obtained by the method of exponential interpolation.

(5) $$FC_{PHEV}=(1-EDR)\times PC_{CS}+EDR\times FC_{CD}\times R$$

In the equation above: *FC_PHEV_* stands for the PHEV fuel consumption conversion (L/100 km); *EDR* stands for electric distance ratio; *PC_CS_* stands for the fuel consumption in CS mode; *FC_CD_* stands for the power consumption in CD mode; *R* is a conversion factor between power consumption and fuel consumption.

### 5.2. Regulation Presumption

Regulation is an important factor in this paper. China is now under the fourth stage of CAFC regulation, and its validity period is before 2020, so is the NEV regulation. In order to predict the relaxation of fuel consumption restriction by 2030, it is necessary to make presumptions about the Dual-credit Regulation between 2021 and 2030.

**Figure 3:**
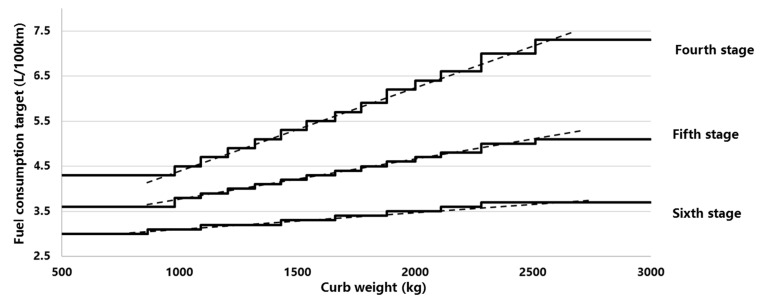
The target value curve of the fifth, sixth, and seventh stages of the CAFC regulation.

The main parameters of the CAFC integral include the target value of each curb weight segment, the multiple numbers for NEV W_i_, and the conversion fuel consumption for NEV FC_BEV_ and FC_PHEV_. Firstly, fit the step-like curve according to the fourth-stage regulation to a linear curve. Then this curve is Shift down 20%. The average target value is 5 L/100 km in 2020, then decreases down to 4 L/100 km in 2025 and 3.2 L/100 km in 2030 [28]. Then, based on the expected new produced vehicle average weight pivot point (1314 kg, 4 L/100 km), the target value curve is rotated clockwise by 40% (the slope is multiplied by 60%), and then, the fifth stage CAFC regulatory target value is fitted according to each mass segment. The gradient curve, the sixth stage curve, is obtained in the same way, and the target value curve of the fourth, fifth, and sixth stages are shown in Figure 3.

The multiple for NEV is set in order to promote the development of the NEV market and is conducive to company compliance. This multiple has been reduced from the fourth stage of the CAFC regulation, from 5 in 2016 to 2 in 2020 [18]. The continued decline of this preferential offer is an inevitable trend in the future in order to eliminate the effect of this parameter on the result. The discount multiple is assumed to be 1 from 2020, which means the multiple of NEV is the same as the fossil-fueled vehicle and then remains unchanged.

In the CAFC regulation, the average conversion fuel consumption of NEV is regarded as zero, which is not reasonable. NEV also generates carbon emissions, which should be considered in the CAFC regulations. Since there is too much preferential offer for the NEV in CAFC regulation, the average conversion fuel consumption of NEV is assumed to be converted on the basis of equal caloric value from the sixth stage. Actually, adopting this method of converting the power consumption is also good for the technology improvement of power consumption. Therefore, the conversion data is presumed and shown in Table 3.

### 5.3. Relaxation of the Fuel Consumption Restriction Quantification

**Figure 4:**
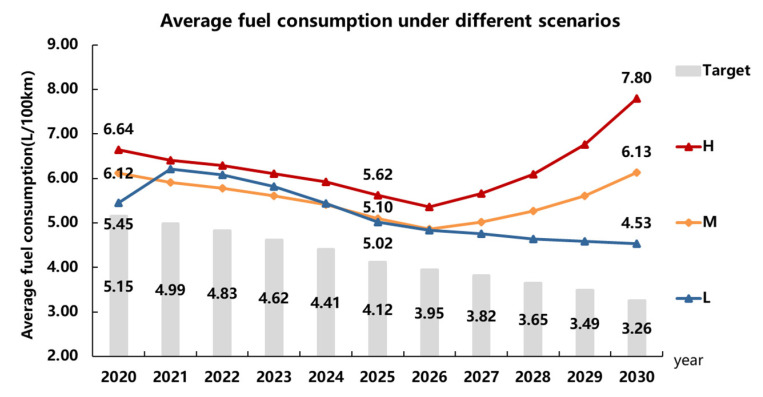
Average fuel consumption under different scenarios.

**Figure 5:**
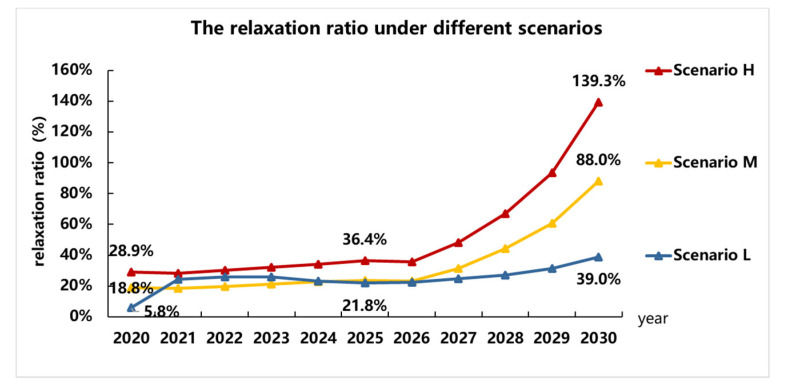
The ratio of the fuel consumption target relaxation.

Based on the market and regulation presumptions above, the fuel consumption target and the actual fuel consumption of the fossil-fueled vehicle in each year under three different scenarios are calculated, as shown in Figure 4. In This paper, the actual fuel consumption value stands for the actual target value of fuel consumption because companies can comply as long as the average fuel consumption is lower than this value. The degree of relaxation can be expressed as the difference between the CAFC target value and the actual target value. The ratio of the fuel consumption relaxation for each year is calculated, as shown in Figure 5.

According to the calculations above, in the Dual-credit Regulation, NEV has a greater impact on fuel consumption restriction, and the development speed of the NEV market is positively related to the degree of relaxation. In scenario “L,” the fuel consumption target in 2030 is relaxed from 3.26 L/100 km to 4.53 L/100 km, and the relaxation ratio is about 39.0%, which is relatively low and controllable. However, in scenario “H,” the relaxation is more severe. In 2020, the CAFC target value will be relaxed from 5.15 L/100 km to 6.64 L/100 km, and the relaxation rate is 28.9%, from 4.12 L/100 km to 5.62 L/100 km in 2025, the relaxation rate is 28.2%. Then in 2030, it is relaxed from 3.26 L/ 100 km to 7.80 L/100 km, and the rate is 139.3%. At this time, the actual target value can be even much higher than the target value in 2018. This results in a “tail-lift” phenomenon, which means for companies, the average fuel consumption of their products could be higher than the year before without violating the Dual-credit Regulation. For the scenario “M” in “The Roadmap,” there is also this “tail-lift” phenomenon. Moreover, since scenario “L” represents the lower bond of the NEV sales volume in NEV regulation, the results between 2021–2024 also show that under the new phase of the NEV regulation, the scenario “M” (lower proportion according to “The Roadmap” prediction) [28] might not be able to meet the NEV regulation. There might be a higher pressure on companies to make enough NEVs for compliance.

There is also a fuel consumption limit for each passenger car model due to the CAFC regulation. This limit depends on the curb weight segment of the model, and this limit is much higher than the target value. A model with fuel consumption higher than its limit is banned from production regardless of whether the manufacturer complies with the Dual-credit Regulation. Therefore, the extremely high average fuel consumption in this study only shows that the restriction of fuel consumption is relaxed due to the calculation method and the credit transaction mechanism. For example, in Figure 4, the 7.80 L/100 km is much higher than the limit of the highest curb weight segment, which shows that the Dual-credit Regulation completely loses the effect of controlling fuel consumption.

**Figure 6:**
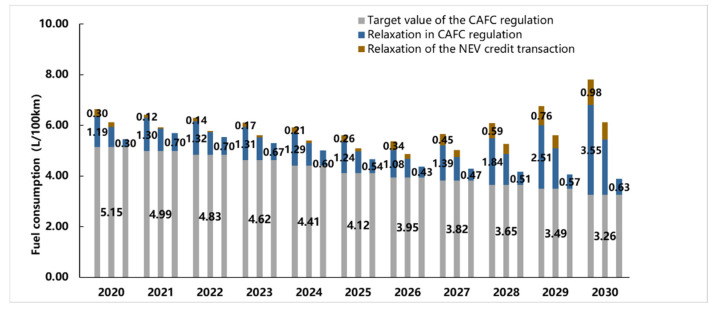
The relaxation effect comparison of the three different factors.

In order to compare the relaxation effect on the fuel consumption restriction between the three factors, the CAFC regulation target value, calculated value of the average fuel consumption in the CAFC regulation, and the actual target value are put together, as shown in Figure 6.

In Figure 6, the “Target value of the CAFC regulation” stands for the average target value of the industry.

The “Relaxation in CAFC regulation” stands for the difference between the CAFC value calculated in the CAFC regulation and the average target value of the industry.

The “Relaxation of the NEV credits compensation” stands for the difference between the actual average fuel consumption of the industry and the CAFC value calculated in the CAFC regulation.

In this Figure, 11 groups of bars are shown representing the results in each year; three bars are included in each group representing the results of “H, M, and L” scenarios, respectively. For clarity, only the data labels in the “H” scenario are shown.

Among the three scenarios of “H,” “M,” and “L.” The fuel consumption is generally controlled in scenario “L” because the sales volume of NEV could barely meet the national minimum requirement of NEV credit ratio, and there is no extra credit to compensate for the lack of CAFC credits. In this scenario, the relaxation of the fuel consumption target is totally due to the preferential for the NEV during the calculation process. In the scenarios “H” and “M,” the relaxation effect is clearly exposed, and the fuel consumption restriction is out of control. In the M and H scenarios, the main factor of the relaxation effect is the preferential offer during the CAFC calculation process before 2025. Then from 2026 to 2030, the effect of the NEV credit compensation is getting stronger. Take scenario “H” as an example, the difference between the actual fuel consumption target value and the target value of the CAFC is 4.53 L/100 km in 2030, and the compensation of NEV credits contributes 0.98 L/100 km, 30% of the total. Therefore, the main reason for the relaxation of fuel consumption target restriction is the preferential offer to NEV during the calculating process. As mentioned above, this offer can be divided into the fuel consumption conversion method of NEV and NEV credit compensation. However, according to the assumptions in this paper, there is no high multiple number W_i_ for NEVs in the CAFC credit calculation process after 2020, which means the relaxation of the restriction is totally due to the conversion method of the power consumption of the NEVs. Even if the conversion method switches to the basic equal caloric value method after 2025, the fuel consumption restriction is still significantly relaxed.

**Figure 7:**
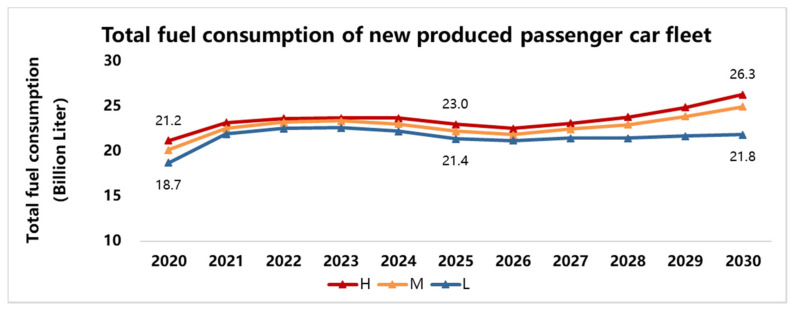
The total fuel consumption of newly produced passenger car fleet.

However, even if the market distribution of NEV develops fast, the total fuel consumption of the passenger car fleet can be controlled to some extent, which is one of the main objectives of the Dual-credit Regulation. The total fuel consumption is roughly estimated with the market growth of passenger cars considered, as shown in Figure 7.

## 6. Conclusions

In general, due to the implementation of the Dual-credit Regulation, there is a trend of the fuel consumption restriction relaxing, and the development speed of the NEV market is positively related to the degree of the relaxation. With a high speed of development, the trend of relaxation may increase significantly after 2025, which will eventually lead to the “tail-lift” phenomenon under the CAFC regulation. At that time, the fuel consumption restriction effect due to the CAFC credit requirement will lose effectiveness.

There are three causes leading to the relaxation of the fuel consumption restriction:

(i). High multiple for NEVs when calculating the corporate average fuel consumption;
(ii). The energy consumption is regarded as 0 during the conversion process;
(iii). The lack of CAFC credits can be compensated by positive NEV credits.

Among them, the main influencing factor is the second one. It is found that even if the equal caloric value method was adopted during the conversion process, the restriction is still significantly relaxed.

The Dual-credit Regulation has a profound impact on the development of China’s passenger cars, and it plays an important role in national fuel-economy technologies and emission reduction goals. The aims of the CAFC credit regulation and NEV credit regulation are similar but independent. The purpose of the CAFC credit regulation is to promote fuel-economy technologies, while the purpose of the NEV credit regulation is to promote the market development of NEV as it is just started.

Dual-credit Regulation is an effective policy that influences the Chinese passenger car industry. In order to promote the development of the Chinese passenger car industry, fuel-economy technologies and the new energy market should be promoted simultaneously. It is suggested that the preferential provision for NEVs in the Dual-credit Regulation should be gradually canceled as the market share of NEV grows. Since the NEV regulation is already established and carried out well, there is no longer a need to keep these preferential provisions. Moreover, the NEV market is developing faster than expected, so extra stimulation is no longer needed. Finally, there should not be any connection between CAFC and NEV regulation, and NEVs should not be considered when calculating the CAFC credit. These suggestions should be well considered during the next phase of the Dual-credit Regulation. Although current regulation is beneficial for the development of the NEV market, the relaxation of the fuel consumption restriction will become obvious after 2025, and a “tail-lift” phenomenon might occur. It is not good for the fuel-economy technique and reducing the dependence on import oil in the long run.

This paper mainly studies the Dual-credit Regulation, and the relaxation effect of fuel consumption restriction is quantified in this research. Additionally, it is found that the proportion of the NEV in the market directly affects the degree of regulation. Moreover, this paper first figures out that the main source of the relaxation is that the energy consumption is regarded as zero during the conversion process. These conclusions are important when formulating the next stage of the Dual-credit Regulation. However, there are still some limitations in this study. HEV (Hybrid electric vehicle) is not well considered in this study because the number of models and sales volume is too small. Some of the provisions have not been considered carefully because with the method proposed in this research, each type of vehicle is regarded as an average vehicle, and thus, the deviation caused by the diversity of technical level may not be considered. The shift of the curb weight with years is not considered. Finally, research based on the data of each corporation should be carried out. Further research needs to be conducted, and the calculating method in this research should be refined.

## Figures and Tables

**Table 1:** The passenger car market hypothesis: market share of NEV. NEV: New Energy Vehicle.

Year	2018	2020	2025	2030
Scenario H	3.8%	10.4%	20%	50%
Scenario M	3.4%	7.0%	15.0%	40%
Scenario L	1.6%	3.8%	7.1%	13.6%

**Table 2:** The hypothesis about NEV market and credits. BEV: battery electric vehicle; PHEV: plug-in hybrid vehicle; NEV: New Energy Vehicle.

Year	2020	2025	2030
BEV/PHEV	2.5	1.5	2.0
Average driving range of BEV	340	380	413
Average credits for one BEV	4.9	1.5	2.0
Credits for each PHEV	2.0	1.6	1.6
Average credits for one NEV	3.2	3.4	3.5

**Table 3:** Power consumption and conversion fuel consumption of NEV. PHEV-CD: charge depleting mode of plug-in hybrid electrical vehicle. PHEV-CS: charge sustaining mode of Plug-in hybrid electrical vehicle.

Year	2018	2020	2025	2030
BEV power consumption (kWh/100 km)	13.4	12.0	10.5	9.6
PHEV-CD power consumption (kWh/100 km)	17.7	15.6	14.0	13.0
PHEV-CS fuel consumption (L/100 km)	6.2	6.0	5.4	5
Driving range of PHEV under CD mode (km)	70	80	80	85
PHEV-EDR (%)	75	90	96	98
BEV fuel consumption conversion (L/100 km)	0	0	1.22	1.12
PHEV fuel consumption conversion (L/100 km)	1.6	1.3	2.50	2.25

## Data Availability

Publicly available datasets were analyzed in this study. This data can be found here: (https://susy.mdpi.com/user/manuscripts/displayFile/ce7daff8e92a0d96701acd4f4ea98422/supplementary (accessed on 1 July 2021)).

## Abbreviations

GHG	Greenhouse gas
ICEV	Internal combustion engine vehicle
PHEV	Plug-in hybrid electrical vehicle
BEV	Battery electrical vehicle
CD	Charge depleting
CS	Charge sustaining
HEV	Hybrid electric vehicle
LCA	Life cycle assessment
CAFC	Corporate Average Fuel Consumption
NEV	New Energy Vehicle
CPCA	China Passenger car Association
OEM	Original Equipment Manufacturer
